# Modulation of Self‐Separating Molecular Catalysts for Highly Efficient Biomass Transformations

**DOI:** 10.1002/chem.202001451

**Published:** 2020-08-13

**Authors:** Lifei Lian, Xiang Chen, Xianfeng Yi, Yubing Liu, Wei Chen, Anmin Zheng, Haralampos N. Miras, Yu‐Fei Song

**Affiliations:** ^1^ State Key Laboratory of Chemical Resource Engineering Beijing Advanced Innovation Center for Soft Matter Science and Engineering Beijing University of Chemical Technology Beijing 100029 P.R. China; ^2^ Wuhan Center for Magnetic Resonance Key Laboratory of, Magnetic Resonance in Biological Systems State Key Laboratory of, Magnetic Resonance and Atomic and Molecular Physics Wuhan Institute of Physics and Mathematics Chinese Academy of Sciences Wuhan 430071 P.R. China; ^3^ WestCHEM School of Chemistry University of Glasgow Glasgow G12 8QQ UK

**Keywords:** acid catalysis, biodiesel, covalent modifications, esterification, polyoxometalates

## Abstract

The energetically viable fabrication of stable and highly efficient solid acid catalysts is one of the key steps in large‐scale transformation processes of biomass resources. Herein, the covalent modification of the classical Dawson polyoxometalate (POMs) with sulfonic acids (‐SO_3_H) is reported by grafting sulfonic acid groups on the POM's surface followed by oxidation of (3‐mercaptopropyl)trimethoxysilane. The acidity of TBA_6_‐P_2_W_17_‐SO_3_H (TBA=tetrabutyl ammonium) has been demonstrated by using ^31^P NMR spectroscopy, clearly indicating the presence of strong Brønsted acid sites. The presence of TBA counterions renders the solid acid catalyst as a promising candidate for phase transfer catalytic processes. The TBA_6_‐P_2_W_17_‐SO_3_H shows remarkable activity and selectivity, excellent stability, and great substrate compatibility for the esterification of free fatty acids (FFA) with methanol and conversion into biodiesel at 70 °C with >98 % conversion of oleic acid in 20 min. The excellent catalytic performance can be attributed to the formation of a catalytically active emulsion, which results in a uniform catalytic behavior during the reaction, leading to efficient interaction between the substrate and the active sites of the catalyst. Most importantly, the catalyst can be easily recovered and reused without any loss of its catalytic activity owing to its excellent phase transfer properties. This work offers an efficient and cost‐effective strategy for large‐scale biomass conversion applications.

## Graphical abstract

**Self‐isolating catalyst**: The covalent modification of the classical Dawson polyoxometalate (POMs) with sulfonic acids (‐SO_3_H) is reported by grafting sulfonic acid groups on the POM's surface followed by oxidation of (3‐mercaptopropyl)trimethoxysilane. The acidity of TBA_6_‐P_2_W_17_‐SO_3_H (TBA=tetrabutyl ammonium) has been demonstrated by ^31^P NMR spectroscopy, clearly indicating the presence of strong Brønsted acid sites. The presence of TBA counterions renders the solid acid catalyst as a promising candidate for phase transfer catalytic processes, during which it self‐separates from the mixture to enable recovery.

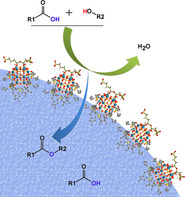

## Introduction

Phase transfer catalysts (PTCs) are widely used in the industrial production of a wide range of chemicals. This is a highly desirable approach as it combines the advantages of both homogeneous and heterogeneous catalytic processes. The advantages of the former include high activity, mild reaction conditions, fast reaction rates, and good accessibility to the catalytic active sites by the substrate;^1^ whereas the latter demonstrates excellent recovery and recycling features.^2^

Polyoxometalates (POMs) are a class of discrete anionic metal oxides of V, Mo, W, etc.^3^ and have been widely used in acid‐catalyzed reactions such as esterification, alkylation, fructose conversion, and hydroxylation of olefins, owing to their highly acidic properties and high thermal stability.^4^ Additionally, the combination of acidic properties, high proton mobility, and stability, render them excellent candidates for the conversions of biomass.^4^ Nevertheless, the low surface area (<10 m^2^ g^−1^) as a solid catalyst, the high solubility in polar reaction media, the ease of agglomeration, and the difficulty of separation significantly limit their application in catalytic reactions. In general, the common strategy employed in these cases is the “immobilization” or “solidification” of catalytically active heteropoly acids (HPAs)^5^ on appropriate supports. For example, in the case of HPA‐immobilized heterogeneous acid catalysts in acid‐catalyzed reactions, different types of supports have been reported, such as silica,^6^ zirconia,^7^ and alumina.^8^ Recently, Juan et al. prepared a series of materials based on immobilizing 12‐tungstophosphoric heteropolyacid on a zirconia support and applied these as the heterogeneous acid catalysts for the esterification of palmitic acid with methanol as a biodiesel model.^7^ Although the immobilization of acid catalysts leads to larger BET (Brunauer–Emmett–Teller) surface areas, improved catalytic activity, and easy separation processes, quite often the immobilization generates a series of other issues such as reduced acid density leading to decreased acidity of the POMs.^5^ An alternative approach could help us overcome these disadvantages, which is the preparation of POM‐based PTCs by careful modulation of the POM‐based catalyst's solubility. The most common strategy to modify the solubility of the catalyst is the careful consideration of the POM's counterions such as alkali and alkali earth metals and their replacement with organic cations such as ionic liquids, quaternary ammonium salts, oligomers, and so on.^9^

It was recently reported that the ionic liquids (IL)–POM systems “IL‐POMs” exhibit high‐density acidic sites and superior catalytic performance in liquid‐phase organic reactions.^10^ For example, Wang et al. synthesized a series of solid non‐conventional IL compounds composed of propane sulfonate functionalized organic cations and heteropolyoxoanions and used them as “reaction‐induced self‐separation catalysts” for various esterification reactions,^11^ even though some mechanical and chemical stability issues and occasionally a negative influence on the acidity of the catalyst may occur.^12^ Moreover, solidification of POMs can be realized by cationic surfactant encapsulation.^13^ For example, Mizuno and co‐workers reported a series of highly efficient POM‐based Lewis acid catalysts containing rare‐earth metals (TBA_6_RE‐POM, TBA=tetrabutyl ammonium, RE = Y^3+^, Nd^3+^, Eu^3+^, Gd^3+^, Tb^3+^, or Dy^3+^) modified with quaternary ammonium salt. In this case, the incorporated rare‐earth metal cation performs as a Lewis acidic site and exhibits significant catalytic properties in the cyanosilylation of ketones and aldehydes.^14^ However, the modification effect of the POMs in PTC systems using quaternary ammonium salts have seldom been investigated in Brønsted acid‐catalyzed reactions. This is due to the fact that the interaction between the organic ammonium cations and the inorganic polyoxoanion is greater than the one between H^+^ and POMs.9e Protons can be easily exchanged with cations, leading to the decrease of the POM's acidic properties.9g

In this work, we report a novel approach which led to the formation of a molecular solid acid catalyst, TBA_6_‐P_2_W_17_‐SO_3_H, by covalent modification of the Dawson polyoxometalate cluster with sulfonic acids (‐SO_3_H). The structural properties and acidity of the TBA_6_‐P_2_W_17_‐SO_3_H are determined by ^31^P NMR spectroscopy, ESI‐MS, X‐ray photoelectron spectroscopy (XPS), and high‐resolution (HR)TEM, etc. Use of the solid catalyst TBA_6_‐P_2_W_17_‐SO_3_H in a range of catalytic biomass transformations revealed superior catalytic activity to the corresponding classical POM archetypes (such as H_3_PW_12_O_40_ and K_10_‐P_2_W_17_) and in some cases even higher than inorganic strong acids such as H_2_SO_4_ under the same reaction conditions. Most importantly, the emulsification effect of the TBA‐modified amphiphilic catalyst induces increased catalytic efficiency in the esterification of oleic acid and methanol owing to effective interactions between substrates and the catalyst. At the end of the reaction, the catalyst self‐separates by precipitation; it can then be easily recovered and reused in multiple catalytic cycles.

## Results and Discussion

**Figure 1:**
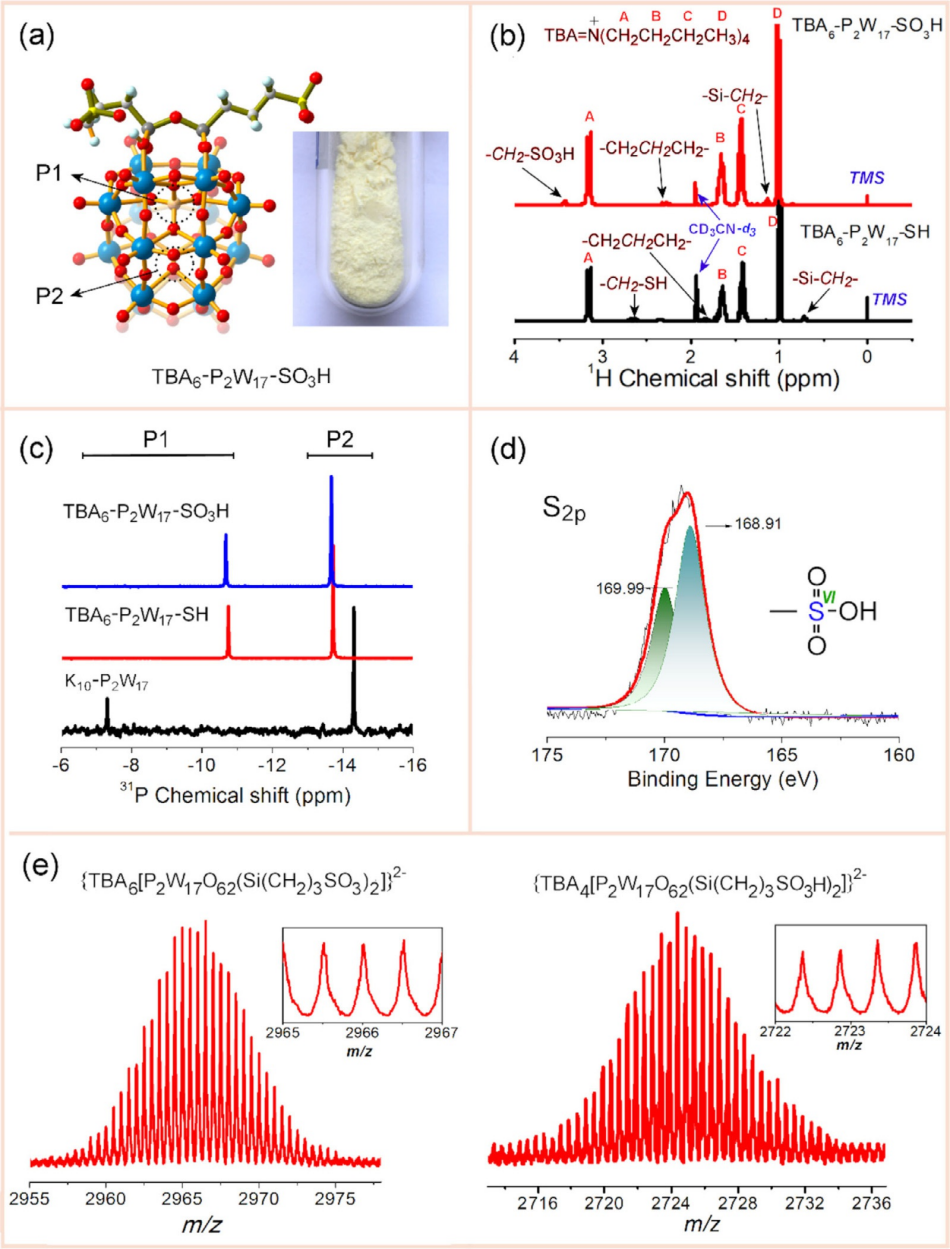
(a) Ball and stick representation of the TBA_6_‐P_2_W_17_‐SO_3_H structure. TBA counterions were omitted for clarity. Color code: P, orange; W, blue; O, red; C, white; S, yellow; Si, gray; H, light blue. Inset: photograph of the catalyst. (b) ^1^H NMR spectra of TBA_6_‐P_2_W_17_‐SH and TBA_6_‐P_2_W_17_‐SO_3_H. (c) ^31^P NMR spectra of K_10_‐P_2_W_17_, TBA_6_‐P_2_W_17_‐SH, and TBA_6_‐P_2_W_17_‐SO_3_H. (d) XPS spectrum of the S 2p core level and (e) ESI‐MS spectra of TBA_6_‐P_2_W_17_‐SO_3_H.

The TBA_6_‐P_2_W_17_‐SO_3_H was obtained through oxidation of the corresponding TBA_6_‐P_2_W_17_‐SH. The light‐yellow powder of TBA_6_‐P_2_W_17_‐SO_3_H was insoluble in water and ethanol, but is readily soluble in CH_3_CN, DMF, and DMSO. As such, the TBA_6_‐P_2_W_17_‐SO_3_H was fully characterized by Fourier transform infrared (FTIR), ^31^P nuclear magnetic resonance (^31^P NMR) spectroscopy, electrospray ionization mass spectra (ESI‐MS), X‐ray photoelectron spectroscopy (XPS), scanning electron microscopy (SEM), high‐resolution transmission electron microscopy (HR‐TEM), high‐angle annular dark field‐scanning transmission electron microscopy (HAADF‐STEM), and thermogravimetric analysis (TGA; Figures S1–8 in the Supporting Information and Figure 1).

The FTIR spectrum of TBA_6_‐P_2_W_17_‐SH (Figure S3 in the Supporting Information) showed the characteristic stretching vibration band of the S−H bond located at 2571 cm^−1^, which disappeared upon oxidation of the starting material. Comparison of the FTIR spectra of the oxidized product and the parent molecule (TBA_6_‐P_2_W_17_‐SH), revealed a set of new bands located at 1043 and 1170 cm^−1^ associated with the stretching vibrations of the C−S and S=O bonds, indicative of the successful oxidation of the ‐SH functional group to ‐SO_3_H. Furthermore, the band centered at 1220 cm^−1^ was attributed to the stretching vibration of the ‐SO_3_H group.^15^

As can be seen from Figure 1 b, the ^1^H NMR spectra of TBA_6_‐P_2_W_17_‐SH and TBA_6_‐P_2_W_17_‐SO_3_H showed the characteristic signals at 1.02, 1.42, 1.65, and 3.15 ppm, corresponding to four kinds of hydrogen atoms associated with the TBA^+^ cation.^13^ The peaks at 0.71, 1.85, and 2.64 ppm for TBA_6_‐P_2_W_17_‐SH can be assigned to the ‐Si‐C*H*_2_‐, ‐CH_2_C*H*_2_CH_2_‐, and ‐C*H*_2_‐SH, which are shifted to 1.13, 2.32, and 3.44 ppm for TBA_6_‐P_2_W_17_‐SO_3_H, respectively. The ^31^P NMR spectra of K_10_‐P_2_W_17_, TBA_6_‐P_2_W_17_‐SH, and TBA_6_‐P_2_W_17_‐SO_3_H show the characteristic two‐line signals. For K_10_‐P_2_W_17_, two ^31^P NMR resonances can be observed at −7.36 and −14.39 ppm^16^ owing to two non‐equivalent phosphorous atoms. In contrast, these resonances are shifted to −10.79 and −13.73 ppm^17^ for the TBA_6_‐P_2_W_17_‐SH and −10.21 and −13.29 ppm for the TBA_6_‐P_2_W_17_‐SO_3_H cluster (Figure 1 c). The downfield resonance can be attributed to the phosphorus close to the organosilyl sites, whereas the upfield resonance was due to the phosphorus atom located close to the W_3_ cap.^17^

XPS study of the TBA_6_‐P_2_W_17_‐SH cluster revealed a band located at 163.5 eV, attributed to the binding energy of the S 2p18a (Figure S5 in the Supporting Information). After oxidation to TBA_6_‐P_2_W_17_‐SO_3_H, the binding energy of the S 2p shifted to higher energy and two closely spaced bands are located at 168.9 and 169.9 eV (Figure 1 d), which can be assigned to two different chemical environments of the covalently grafted ‐SO_3_H groups. The observed increase of the binding energy in the XPS spectrum indicates a decrease in electron density on the sulfur atom.18b The binding energy observed in the case of the TBA_6_‐P_2_W_17_‐SO_3_H cluster appears to be higher owing to the more electronegative oxygen atoms on the POM shell adjacent to the ‐SO_3_H group compared with conventional catalyst materials such as SiO_2_‐SO_3_H.15b The ESI‐MS helped us confirm the composition of the synthesized cluster as well as its relevant stability in the relevant solvent medium.^19^ The ESI‐MS spectrum revealed a complex isotope pattern (Figure S6, Table S1 in the Supporting Information) and all of the signals can be clearly assigned. The isotopic distribution envelopes of the intact [TBA_6_‐P_2_W_17_‐SO_3_]^2−^ and [TBA_4_‐P_2_W_17_‐SO_3_H]^2−^ cluster were located at *m*/*z*=2967.2 and 2726.0, respectively (Figure 1 e).

SEM images of TBA_6_‐P_2_W_17_‐SO_3_H showed irregular particles, which were uniformly distributed (Figure S8 a in the Supporting Information) with a diameter ranging from 30 to 50 nm. HRTEM images of TBA_6_‐P_2_W_17_‐SO_3_H (Figure S8 b in the Supporting Information) exhibited homogeneously distributed dark dots of approximately 1 nm in diameter,^20^ which can be ascribed to the POM clusters. HAADF‐STEM of the as‐prepared TBA_6_‐P_2_W_17_‐SO_3_H sample indicated the presence of W, P, O, S, and Si elements (Figure S8 c in the Supporting Information).

**Figure 2:**
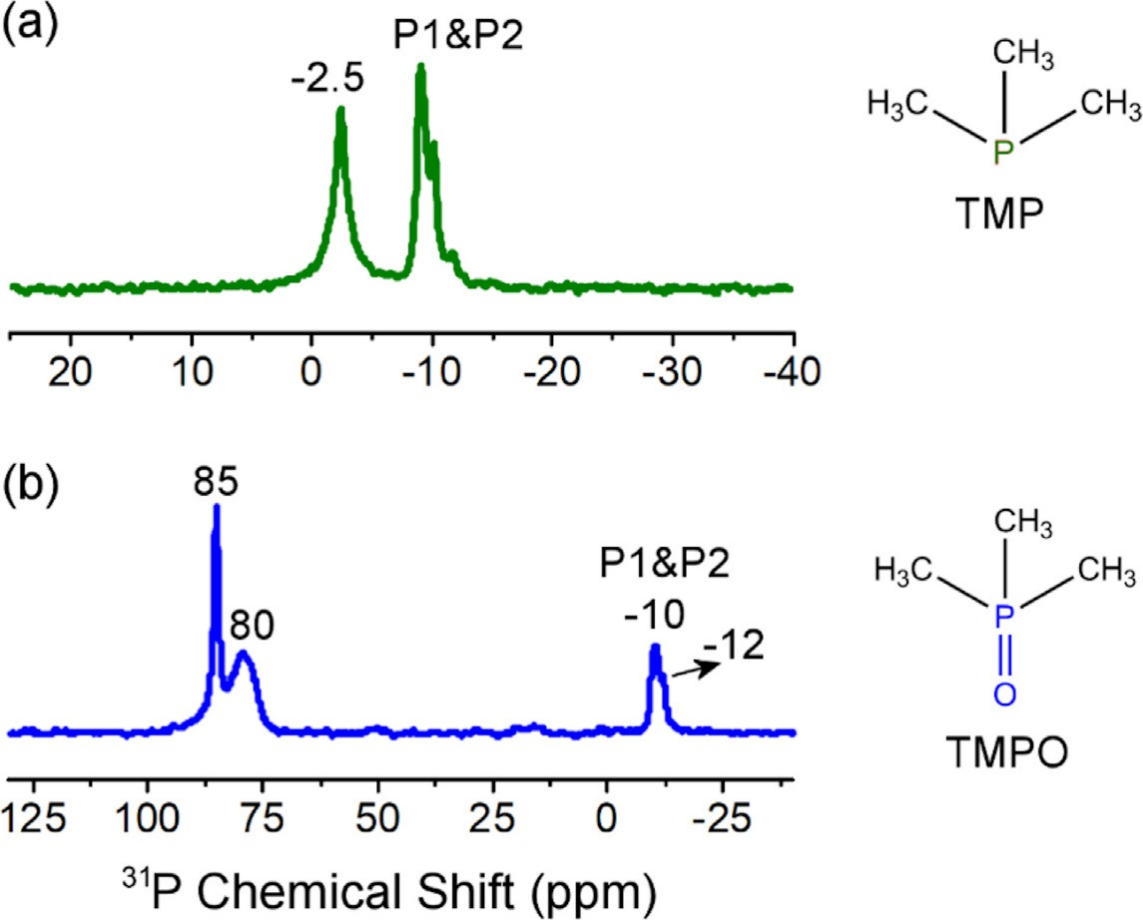
^31^P MAS NMR spectra of (a) TMP and (b) TMPO adsorbed on sample TBA_6_‐P_2_W_17_‐SO_3_H.

Acid–base titrations were employed to analyze the acidic groups quantitatively (Table S2 in the Supporting Information). As determined by using the Hammett indicators, the TBA_6_‐P_2_W_17_ cluster gave an *H*_0_ value > −0.2 whereas the corresponding value in the case of the TBA_6_‐P_2_W_17_‐SO_3_H cluster was found to be < −11.4 (Table S2 in the Supporting Information), which was comparable to that of the concentrated H_2_SO_4_ (*H*_0_=−11.9).^21^ As such, the acidity of TBA_6_‐P_2_W_17_‐SO_3_H was higher than that of the non‐modified cluster, TBA_6_‐P_2_W_17_. Furthermore, the acid properties of TBA_6_‐P_2_W_17_‐SO_3_H were characterized by ^31^P MAS (magic angle spinning) NMR probe techniques involving adsorbed trimethylphosphine (TMP) and trimethylphosphine oxide (TMPO), which is a sensitive and reliable approach to determine the type of acidity (Brønsted or Lewis acid) and the acid strength of solid acid catalysts.^22^ As shown in Figure 2, the ^31^P resonance at −2.5 ppm of adsorbed TMP confirmed the Brønsted acidity of the TBA_6_‐P_2_W_17_‐SO_3_H cluster. Moreover, the strength of the Brønsted acidity was explored by TMPO adsorption, where two ^31^P resonance peaks centered at 85 and 80 ppm clearly indicate the presence of Brønsted acid sites with different acid strengths (Figure 2). As the threshold *δ* ^31^P value of TMPO for superacidity was demonstrated to be approximately 86 ppm (with an acid strength similar to 100 % H_2_SO_4_),^23^ it can be concluded that the TBA_6_‐P_2_W_17_‐SO_3_H modified cluster exerted superacidity, which may facilitate a superior catalytic performance.

**Figure 3:**
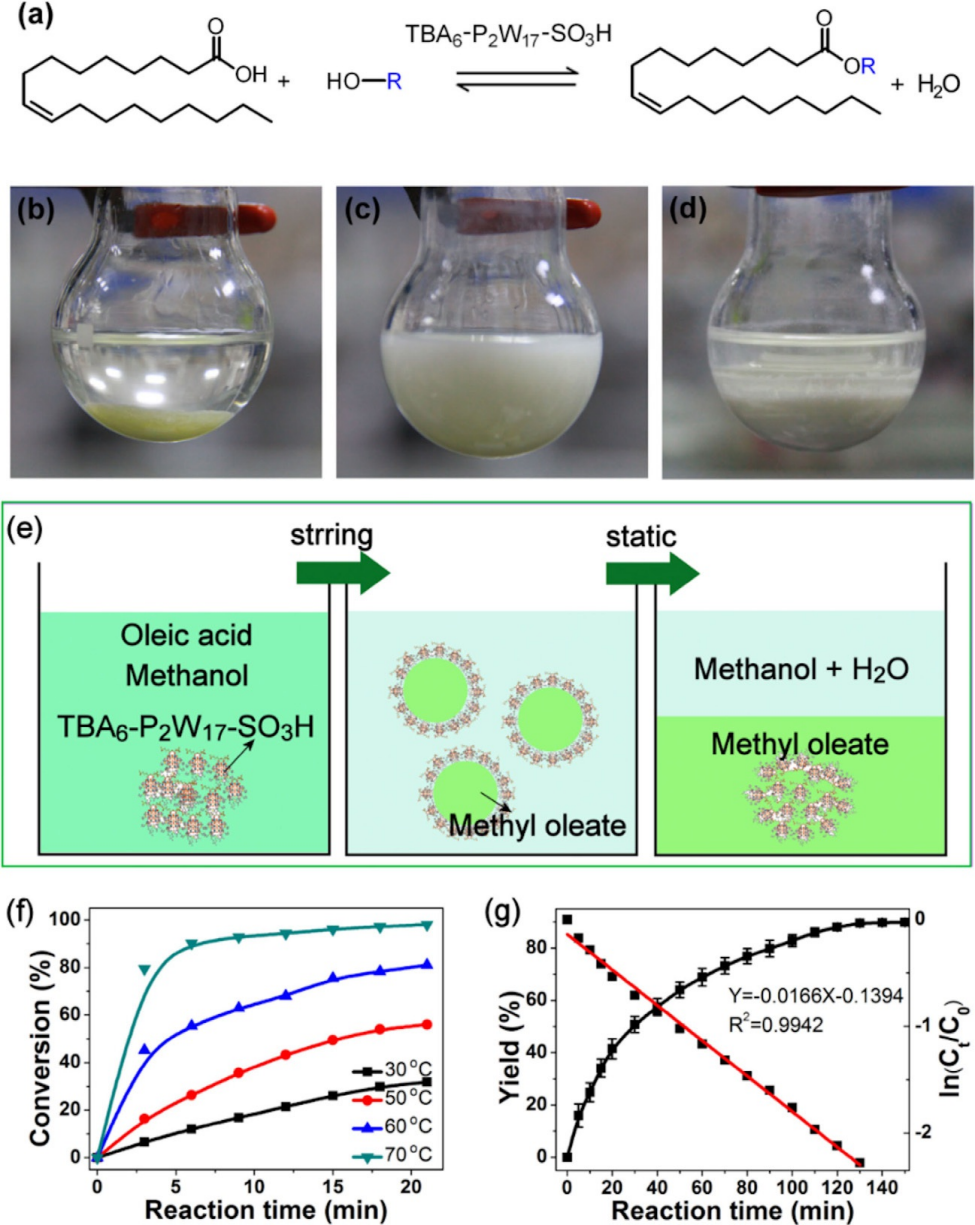
(a) The reaction scheme of the catalytic reaction between oleic acid and methanol. (b) TBA_6_‐P_2_W_17_‐SO_3_H (light‐yellow solid at the bottom of the flask) and oleic acid were added to the reaction flask at the beginning of the reaction. (c) The reaction mixture gradually became turbid, forming an emulsion as a function of the time. (d) The catalyst precipitated at the end of the reaction. (e) Schematic representation of the catalytic process. (f) Optimization of temperature effect on the esterification of oleic acid and methanol by TBA_6_‐P_2_W_17_‐SO_3_H; reaction conditions: oleic acid (2 mmol), methanol (20 mmol), TBA_6_‐P_2_W_17_‐SO_3_H (56.7 mg, 10 wt % based on the weight of oleic acid), 70 °C. (g) The esterification reaction kinetic profiles of oleic acid and methanol by TBA_6_‐P_2_W_17_‐SO_3_H; reaction conditions: oleic acid (2 mmol), methanol (20 mmol), TBA_6_‐P_2_W_17_‐SO_3_H (5.7 mg, 1 wt % based on the weight of oleic acid) at 70 °C.

Based on the above observations, we explored the catalytic efficiency of the modified catalyst in the esterification reaction of oleic acid with methanol as it is a very important pretreatment step in the production of biodiesel from high free fatty acid feedstocks (Figure 3 a). During the course of the catalytic reaction, the generation of the emulsion owing to the presence of the amphiphilic molecule proved to be beneficial for the catalytic performance owing to improved interaction of the substrate with the catalytic sites of the POM derivative. We investigated the phase transition during the reaction in the presence of the reactant organic matrix and our modified catalyst TBA_6_‐P_2_W_17_‐SO_3_H. At the beginning of the reaction, oleic acid and methanol were mixed, to which the TBA_6_‐P_2_W_17_‐SO_3_H was added as a light‐yellow solid (Figure 3 b) generating a heterogeneous mixture. Interestingly, as a function of time, the solution became gradually turbid (Figure 3 c), and a stable emulsion was formed. The emulsion was developed as a result of the formation of hydrophobic POM‐based micelles containing the product of the catalytic reaction as depicted schematically in Figure S10 (in the Supporting Information). As the catalytic reaction progressed, the micelles became unstable, leading to separation of the reaction mixture into two liquid phases and subsequent precipitation of the catalyst as a white powder (Figure 3 d). The phase separation and regeneration of the heterogeneous system induced the separation of the solid catalyst as well as the phase containing the final product of the catalytic reaction. Overall, the TBA_6_‐P_2_W_17_‐SO_3_H cluster proved to be a very efficient catalyst, giving an excellent yield and selectivity of 98.7 and 99.0 %, respectively, at 70 °C in 20 min, which appeared to be largely enhanced compared with other examples reported so far.^24^, ^25^, ^26^, ^27^, ^28^, ^29^

To determine the optimum reaction conditions, we studied the effect of the reaction temperature and time on the esterification of oleic acid with methanol (Figure 3, Figures S11, S12 in the Supporting Information). Generally, the yield of methyl oleate increased as a function of time. In 3 min, the methyl oleate yield increased slowly to 16.5 % at 50 °C, and it increased quickly to 79.5 % at 70 °C. In 20 min, the yield of methyl oleate could reach 31.8 % at 30 °C, 57.0 % at 50 °C, 81.0 % at 60 °C, and 98.7 % at 70 °C,.

**Figure 4:**
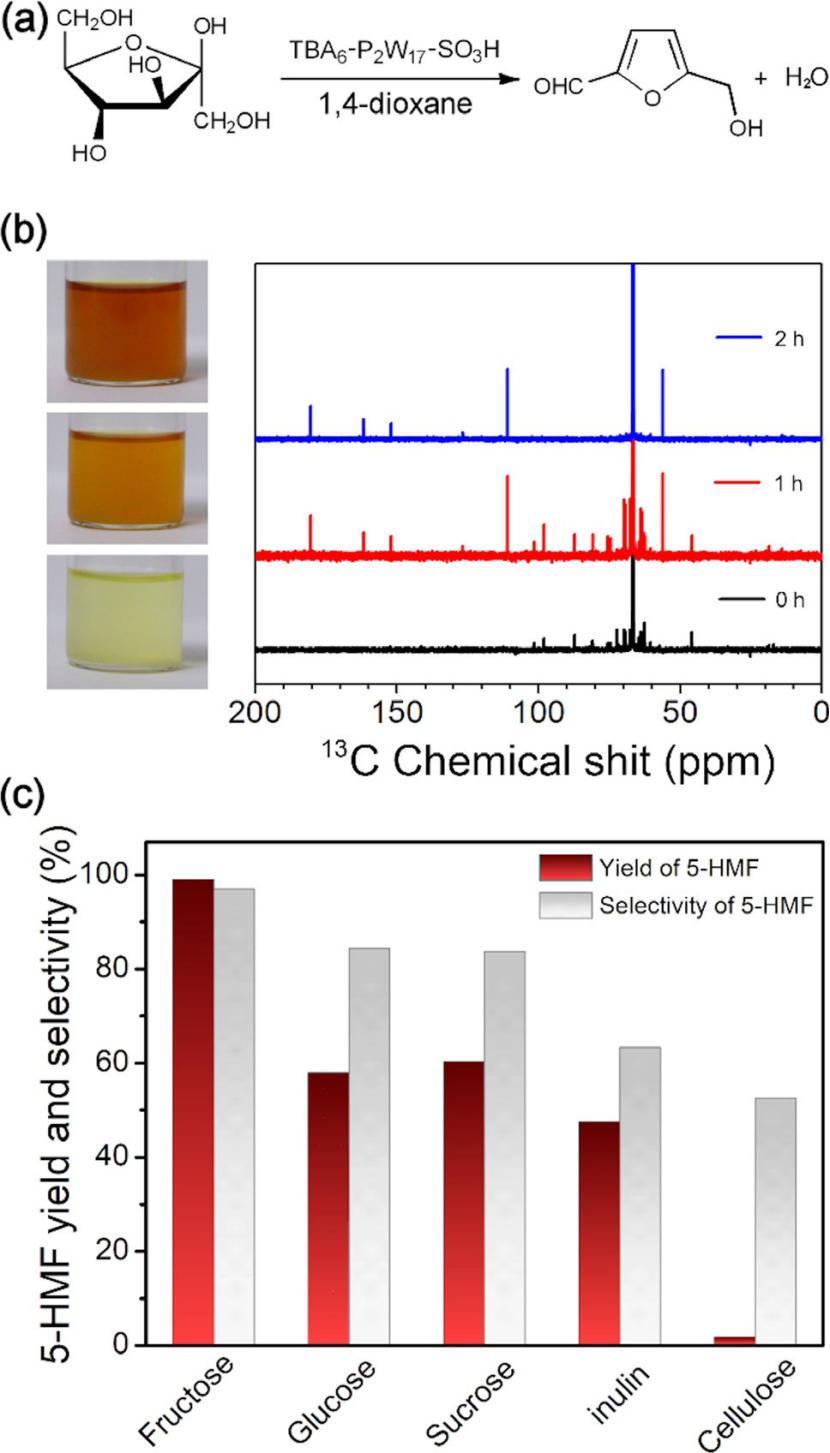
(a) The catalytic transformation of fructose to 5‐HMF. (b) The ^13^C NMR spectra of fructose dehydration by TBA_6_‐P_2_W_17_‐SO_3_H in 1,4‐dioxane at 100 °C. (c) Catalytic conversion of various carbohydrates over TBA_6_‐P_2_W_17_‐SO_3_H. Conditions: carbohydrates (0.45 g), 1,4‐dioxane (10 mL), and TBA_6_‐P_2_W_17_‐SO_3_H (0.15 g), *t*=2 h.

The yield of methyl oleate and ln(*C_t_*/*C*_0_) were plotted against the reaction time as shown in Figure 4 g, in which *C*_0_ and *C_t_* are the initial oleic acid concentration and concentration at time *t*, respectively. The linear fit of the data revealed that the catalytic reaction exhibited a pseudo‐first‐order kinetic constant for the esterification reaction (*R* ^2^=0.9942). The rate constant *k* of the conversion of oleic acid was determined to be 0.0166 min^−1^ based on Equations (1), (2).

(1) $$-dC_{t}/dt=k$$

(2) $$\ln(C_{0}/C_{t})=kt$$

The above results obtained from our system along with data of previously reported catalysts are summarized in Table S4 (in the Supporting Information). The H_3_PW_12_O_40_ and H_3_PMo_12_O_40_ acting as homogeneous catalysts in this reaction revealed lower yields of 67.7 and 65.7 % (entries 2 and 3, Table S4 in the Supporting Information), whereas H_2_SO_4_ showed a high yield of 88.3 % (entry 1, Table S4 in the Supporting Information). Compared with the H_3_PW_12_O_40_, H_3_PMo_12_O_40_, and H_2_SO_4_ catalysts reported so far,^29^, ^30^ esterification hardly occurred in the presence of the K_10_‐P_2_W_17_, TBA_6_‐P_2_W_17_, and TBA_6_‐P_2_W_17_‐SH catalysts under the employed conditions. The relevant yield of the methyl oleate in this case was found to be only 0.6, 1.0, and 0.7 %, respectively (entries 4–6, Table S4 in the Supporting Information). In marked contrast, the presence of the modified TBA_6_‐P_2_W_17_‐SO_3_H catalyst induced a self‐separating liquid–solid heterogeneous reaction system and demonstrated a superior yield of 98.7 % (entry 7, Table S4 in the Supporting Information). The observed efficiency of the modified catalytic system clearly outperforms the one observed in the case of the non‐modified adduct (TBA_6_‐P_2_W_17_) as well as the top performing examples reported previously.

Table 1 summarizes the conditions and the catalytic performance of different catalysts used for the catalytic esterification reaction. It is evident that the modified TBA_6_‐P_2_W_17_‐SO_3_H catalyst revealed a high conversion rate with a turnover frequency (TOF) of 52.8 h^−1^ and 546.0 h^−1^ at 298 and 343 K, respectively (entries 8 and 9, Table 1). The grafting of sulfonic acid (SO_3_H) functional groups on the POM shell modified the acidity of the catalyst, which clearly benefited the catalytic efficiency.

**Table 1:** Catalytic performance of different catalysts in the esterification of oleic acid with methanol.

Entry	Catalyst	Catalyst amount [wt %/mmol^[c]^]	*T* [K]	Acid/MeOH [mmol]	TON^[d]^	TOF [h^−1^]^[e]^	Ref.
1	H_3_PW	4.0/0.01	298	1:10 (1.0)	73.4	3.7	[24]
2	PzS‐PW	8.9/0.01	298	1:10 (1.0)	75.9	3.8	[24]
3	*p*‐TSA	3.0/0.03	333	1:3 (6.0)	–	230.4	[25]
4	2‐Ce‐ZrO_2_/TiO_2_‐SO_4_^2−^‐600	5.0/4.41	348	1:6 (35.4)	–	4.6	[26]
5	10 % SZ‐MIL‐101	11.0/0.20	338	1:77 (3.2)	–	15.6	[27]
6	GO‐S	0.5/0.26	338	1:22 (70.8)	–	304.6	[28]
7	SO_4_^2−^/Sr‐Fe oxide‐4	10.0/0.26	373	1:4 (–)	–	138.6	[29]
8	TBA_6_‐P_2_W_17_‐SO_3_H^a^	10.0/0.03	298	1:10 (2.0)	8.8	52.8	this work
9	TBA_6_‐P_2_W_17_‐SO_3_H^b^	10.0/0.03	343	1:10 (2.0)	9.1	546.0	

[a] Reaction conditions: oleic acid 2 mmol, methanol 20 mmol, catalyst 56.7 mg (10 wt % based on the weight of oleic acid), 25 °C. [b] Reaction conditions: oleic acid 2 mmol, methanol 20 mmol, catalyst 56.7 mg (10 wt % based on the weight of oleic acid), 70 °C. [c] Calculated from the content of S, ‐SO_3_H, or acid content. [d] The turnover number (TON) is based on the esterification product (mol) produce per molar acid site in the catalyst. [e] The turnover frequency (TOF) is based on the esterification product (mol) produced per hour and per molar acid site in the catalyst. *p*‐TSA: *p*‐toluenesulfonic acid; PzS‐PW: sulfonic acid‐functionalized pyrazinium phosphotungstate; 2‐Ce‐ZrO_2_/TiO_2_‐SO_4_^2−^‐600: 2 and 600 represent the Ce concentration (wt %) and calcination temperature (°C), respectively; 10 % SZ‐MIL‐101: sulfated zirconia/metal–organic framework; GO‐S: sulfur‐rich graphene oxide; SO_4_^2−^/Sr‐Fe oxide‐4: sulfated strontium‐ferric oxide (Sr/Fe atomic ratio of 34.58).

To investigate further the general applicability of the TBA_6_‐P_2_W_17_‐SO_3_H catalyst in esterification reactions, a series of various combinations of fatty acid and alcohol substrates were evaluated. Table 2 and Table S5 (in the Supporting Information) summarize the findings of this effort. More specifically, for small molecular weight alcohols such as methanol, ethanol, propanol, butanol, and pentanol, the yield of the esterification reaction usually reached a value of more than 97 % within 90 min (entries 1–5, Table 2). The time required to reach a yield of 97 % increased according to the increase of the alcohol′s molecular weight. On the other hand, with the use of small molecular acids, such as propionic, butyric, valeric, and caprylic acid, the esterification reactions proceeded rapidly, reaching more than 97 % in 30 min (entries 6–10, Table 2). Interestingly, equally excellent catalytic activity and selectivity were obtained in the esterification of long‐chain acids and methanol (Figure S14 in the Supporting Information) as demonstrated in the synthesis of benzyl laurate, benzyl hexanoate, methyl 5‐hexanoate, and methyl methacrylate (entries 11–14, Table 2). These results demonstrated the general applicability of the modified TBA_6_‐P_2_W_17_‐SO_3_H acid catalyst in the esterification of a variety of acids and alcohols for the production of biodiesel.

**Table 2:** Results of various esterification reactions over TBA_6_‐P_2_W_17_‐SO_3_H.

Entry	Carboxylic acid	Alcohols	Yield [%]	*t* [min]
1	oleic acid	methanol	98.67	20
2	oleic acid	ethanol	99.23	30
3	oleic acid	propanol	96.75	65
4	oleic acid	butanol	97.12	75
5	oleic acid	pentanol	97.57	90
6	propionic acid	methanol	97.64	20
7	butyric acid	methanol	97.41	25
8	valeric acid	methanol	97.16	25
9	caproic acid	methanol	97.46	25
10	heptylic acid	methanol	97.67	30
11	lauric acid	benzyl alcohol	96.59	120
12	caproic acid	benzyl alcohol	97.62	120
13	5‐hexinic acid	methanol	98.83	30
14	methacrylic acid	methanol	97.42	30

Reaction conditions: acid 2 mmol, alcohol 20 mmol, catalyst 10 wt % based on the weight of oleic acid, 70 °C.

In an effort to investigate the recyclability of the TBA_6_‐P_2_W_17_‐SO_3_H, the catalyst was separated by filtration after the first run, washed with methanol, and dried under vacuum before use in the next catalytic cycle. The yield of methyl oleate decreased slightly from 98.67 to 94.35 % after five successive runs, whereas negligible loss of reactivity could be detected. In addition, the ^31^P NMR, XPS, and elemental (C, N, O, P, Si, S, and W) mapping data obtained for the recycled catalyst were found to be the same as that of the fresh one, which is indicative of the structural stability during the course of the catalytic cycles (Figure S15 in the Supporting Information).

5‐Hydroxymethylfurfural (5‐HMF) is a potentially promising platform molecule that can be converted into several valuable chemicals, including 2,5‐dimethylfuran, 2,5‐diformylfuran, 1,6‐hexanediol, formic acid, and levulinic acid.^31^ Considering the efficiency observed in the esterification reactions, we investigated the potential use of TBA_6_‐P_2_W_17_‐SO_3_H in the catalytic transformation of different carbohydrates into 5‐HMF.

In this case, a series of different organic solvents were evaluated for their potential effect on the fructose dehydration at 100 °C (Figure S16 in the Supporting Information). 1,4‐Dioxane proved to be the most effective solvent medium, reaching a yield of 99.0 % for the production of 5‐HMF at 100 °C in 2 h, whereas the obtained yields when using DMSO, DMF, methanol, ethanol, and water as solvents were the 94.9, 88.5, 1.5, 39.1, and 2.8 %, respectively. Furthermore, the effect of the reaction temperature (Figure S17 in the Supporting Information) and catalyst dosage (Figure S18 in the Supporting Information) on the catalytic activity of fructose dehydration were investigated and they optimum values found to be 100 °C and 150 mg, respectively. It should be noted that TBA_6_‐P_2_W_17_‐SO_3_H showed improved catalytic conversion than the one observed in the case of strong inorganic acids such as H_2_SO_4_ 32 and HCl.^33^

To improve further our understanding of the fructose dehydration reaction, we monitored the catalytic reaction using ^13^C NMR spectroscopy. As shown in Figure 4 b, at the beginning of the catalytic reaction, the signals located in the range 50–120 ppm can be assigned to the cyclic forms of fructose (the 68.5 ppm peak corresponds to the 1,4‐dioxane solvent).^34^ A decrease of the signal's intensity corresponding to the fructose molecules was observed as a function of the time, whereas new peaks gradually appeared at 180.4, 161.5, 152.0, 126.7, 111.0, and 56.1 ppm, which can be assigned to the production of 5‐HMF.^35^ Finally, ^13^C NMR spectroscopy revealed the complete transformation of the fructose within a period of 2 h, during which the only detectable products in the reaction mixture were 5‐HMF and 1,4‐dioxane solvent. During the catalytic transformation of fructose, the color of the reaction mixture turned gradually from colorless to orange‐yellow. Catalytic recycling experiments showed the decrease of 5‐HMF yield from 94.9 to 90.2 % after four consecutive runs, indicating minor leaching of the catalyst (Figure S19 in the Supporting Information).

The broad utility of the catalyst was further demonstrated by investigating the efficiency during the catalytic transformation of different substrates (Figure 4 c) over TBA_6_‐P_2_W_17_‐SO_3_H in 1,4‐dioxane. Using a wide range of carbohydrates as substrates such as glucose, sucrose, and inulin, we were also able to obtain decent yields of 57.9, 60.3, and 47.5 %, during their catalytic transformation to 5‐HMF. However, only 1.6 % of HMF product was obtained when cellulose was used as the substrate. This observation is indicative of the catalyst's high efficiency and selectivity in the case of monosaccharides or disaccharides but poor performance in the case of polysaccharide substrates. It is worth noting that the difference in yields observed for the dehydration of glucose (57.9 %) and fructose (99.0 %) could be due to the lack of co‐existence of Brønsted (B) and Lewis (L) acidic sites in the catalytic system, which seem to be required for the efficient transformation of glucose or cellulose to HMF.^36^

## Conclusion

The covalent tethering of sulfonic acids on the shell of the Dawson cluster was achieved by surface grafting and oxidation of (3‐mercaptopropyl)trimethoxysilane. The employed approach led to the modulation of the Brønsted acidity of this self‐separating phase transfer molecular catalyst, which exhibits superior performance in biomass transformations owing to its superacidic properties. The acidity of the catalyst was determined by Hammett indicators, potentiometric titration, and ^31^P MAS NMR spectroscopy, confirming its approximate superacidity. The modified molecular catalyst, TBA_6_‐P_2_W_17_‐SO_3_H, showed excellent catalytic activity and selectivity in a wide range of acid‐catalyzed reactions, such as the esterification of oleic acid with a yield of 99.0 %. Interestingly, the emulsification effect of the modified amphiphilic catalyst not only induced an increased catalytic efficiency during the catalytic transformation of the substrates owing to the homogeneity of the system but also led to a self‐separating catalytic system at the end of the catalytic cycle owing to the destabilization of the emulsion and self‐precipitation of the catalyst. The embedded emulsification–precipitation cycle induces excellent self‐recycling properties to the catalytic system, leading to facile and low‐cost recovery of the catalyst at high yields. The design approach described herein paves the way for further development of cost‐effective highly efficient solid acid catalysts engineered for targeted catalytic transformations of biomass‐derived raw materials to high value‐added chemicals.

## Conflict of interest

The authors declare no conflict of interest.

## Supporting information

As a service to our authors and readers, this journal provides supporting information supplied by the authors. Such materials are peer reviewed and may be re‐organized for online delivery, but are not copy‐edited or typeset. Technical support issues arising from supporting information (other than missing files) should be addressed to the authors.

Supplementary
