# Supplementary material for: Modulation of Self‐Separating Molecular Catalysts for Highly Efficient Biomass Transformations
Source: Chemistry. 2020 Aug 13;26(51):11900–8. doi: 10.1002/chem.202001451 (PMC7540606; doi:10.1002/chem.202001451)
Supplement: Supplementary file 1 — Supplementary [file CHEM-26-11900-s001.pdf]

Chemistry–A European Journal

Supporting Information

Modulation of Self-Separating Molecular Catalysts for Highly Efficient Biomass Transformations

Lifei Lian+,[a] Xiang Chen+,[a] Xianfeng Yi,[b] Yubing Liu,[a] Wei Chen,*[a] Anmin Zheng,[b] Haralampos N. Miras,*[c] and Yu-Fei Song*[a]

EXPERIMENTAL DETAILS

1. Chemicals and regents.

Orthophosphoric acid (H_3PO_4, ≥85%), bromine, ammonium chloride, potassium chloride, potassium hydrogen carbonate, hydrochloric acid, tetrabutylammonium bromide (TBA·Br), hydrogen peroxide (30 wt%), ethyl ether, dibasic sodium phosphate, sodium carbonate, sodium metasilicate, sodium tungstate, toluene (≥99.5%) and sulfuric acid were purchased from Beijing Chemical Reagent Factory and used as received without further purification. (3-Mercaptopropyl)trimethoxysilane ($C_6H_{16}O_3SSi$, 97%) acetonitrile, acetonitrile-d_3, acetic acid (≥99%), dodecanoic acid (99%), myristic acid (99%), palmitic acid (95%), octadecanoic acid (98%), n-propionic acid (≥99.5%), n-butyric acid (≥99.5%), n-valeric acid (≥99.5%),n-caproic acid (≥99.5%), n-heptanoic acid (≥99.5%), n-caprylic acid (≥99.5%), adipic acid (99%), lauric acid (99%), oleic acid (90%), benzoic acid (99%), methyl alcohol (99.5%), ethanol (99.5%), n-propanol (≥99.5%), isopropanol, n-butyl alcohol (≥99.5%), n-pentanol (≥99.5%), n-hexanol (≥99.5%), n-heptanol (≥99.5%), n-octyl alcohol, 1,3-propanediol(98%), phenethyl alcohol (>98%), 5-hexynoic acid, methacrylic acid were purchased from Alpha Aesar and used as received without further purification. Hammet indicator solution (0.1 wt%): 2-Nitroaniline (99%, pka = –0.2), 2,4-Dinitroaniline (98%, pka = –4.4), 2-Benzoylnaphthalene 2-bromo-4 (99%, pka = –5.9), 6-Dinitroaniline (98%, pka = –6.6), Antraquinone (98%, pka = –8.1), 4-Nitrotoluene (99%, pka= -11.4), 4-Fluoronitrobenzene (99%, pka= -12.4) were purchased from Energy Chemical and used as received without further purification.

2. Synthesis of POMs.

The $K_6[\alpha\text{-}P_2W_{18}O_{62}]\cdot xH_2O$,[S1] $K_{10}[\alpha\text{-}P_2W_{17}O_{61}]\cdot 20H_2O$,[S2] $TBA_6\text{-}P_2W_{17}$[S3] and $TBA_6\text{-}P_2W_{17}\text{-}SH$[S4] were prepared according to previously reported literature method. 0.3 g of $TBA_6\text{-}P_2W_{17}\text{-}SH$ was suspended in 9 mL of 30 wt% H_2O_2. The suspension was stirred at room temperature for 12 h. After the oxidation treatment, the resulting solution was filtered and washed with methanol. The wet material was suspended (1 wt%) in 1 M H_2SO_4 solution and stirred for 12 h. Finally, the material was washed several times by methanol and dried at 333 K

under vacuum overnight. Yield: 0.259 g, 86.3%. ^{1}H NMR (CD_3CN): δ = 1.02 [t, 72H, -CH_2*CH_3* (TBA)], 1.13 (t, 4H, -Si-*CH_2*-), 1.42 [m, 48H, -CH_2*CH_2*CH_3 (TBA)], 1.65 [m, 48H, -CH_2*CH_2*CH_2- (TBA)], 2.32 (m, 4H, -CH_2*CH_2*CH_2-), 3.15 [t, 48H, N-*CH_2* (TBA)], 3.44 (t, 4H, –*CH_2*-SO_3H) ppm. FT-IR (KBr, cm^{-1}): 2963, 2869, 1220, 1170, 1085, 1043, 1026, 943, 905, 806. ESI-MS (negative; MeCN as solution): *m/z* = 2726.0 $[M\text{-}2TBA]^{2-}$. ^{31}P NMR (CD_3CN): δ = -10.72, -13.73 ppm. $C_{102}H_{230}O_{68}N_6S_2P_2Si_2W_{17}$ (5936.45): calcd. C 20.62, N 1.41, H 3.87, S 1.08; found C 20.57, N 1.38, H 3.81, S 1.12.

3. Analytical techniques.

Fourier transform infrared (FT-IR) spectra were carried out on a Bruker Vector22 infrared spectrometer using KBr pellet method. The ^{1}H Nuclear Magnetic Resonance (^{1}H NMR), ^{13}C Nuclear Magnetic Resonance (^{13}C NMR), ^{31}P Nuclear Magnetic Resonance (^{31}P NMR) spectroscopy were obtained on a Bruker AV400 NMR spectrometer at resonance frequency of 400 MHz. The electrospray ionization mass spectrum (ESI-MS) was recorded using a Xevo G2 QT ESI-MS, and all experiments were performed in negative mode using acetonitrile as solvent. C, H, N and S analyses were carried out on Vario EL in Peking University. X-ray photoelectron spectroscopy (XPS) measurements were performed with monochromatized AlKa exciting X-radiation (PHI Quantera SXM). Scanning electron microscopy (SEM) images analytical data were obtained using a Zeiss Supra 55 SEM equipped with an EDX detector. High-resolution transmission electron microscopy (HR-TEM) images were obtained on a JEM 3010 electron microscope equipped with an energy dispersive X-ray (EDX) detector. Elemental Analyses were carried out using varioEL cube from Elementar Analysensysteme GmbH. High-angle annular dark field-Scanning transmission electron microscopy (HAADF-STEM) measurements were obtained using a Titan 80-300 at 80 kV and a FEI Tecnal G2 F20 U-TWIN instrument at an operating voltage of 200 kV. Thermogravimetric analysis (TGA) was acquired using a TG/DSC 1/1100 SF using a METTLER TOLEDO under nitrogen atmosphere at a heating rate of 10 ^{o}C /min. The catalytic reaction was analyzed on an Agilent 7890A gas chromatograph (Manufactured by Agilent Co. Ltd.) using a flame ionization

detector (GC-FID). A HP-5 capillary column (30 m length and 0.32 mm inner diameter) was used for separation. N_2 was used as carrier gas at a flow rate of 5 mL/min. The oven temperature was initially set at 140 ^oC for 0.5 min, then increased to 210 ^oC with a ramp rate of 20 ^oC /min, and kept at 250 ^oC for 5 min. Then increased to 270 ^oC using a ramp rate of 10 ^oC /min. The fructose was analyzed on Agilent Hi-Plex Ca column equipped with refractive index detector. The content of 5-HMF was analyzed by an Agilent 1260 HPLC (UV wavelength, 284 nm; C18 column 5 μm; 250 × 4.6 mm), using 60% methanol in ultra-pure water as mobile phase at a flow rate of 0.9 $mL·min^{-1}$. The column temperature was maintained at 35 ^oC .

4. The solid state ^{31}P.MAS NMR spectra

All the NMR experiments were performed on a Bruker AVANCE-III 500 MHz spectrometer at Larmor frequencies of 202.63 MHz for ^{31}P nucleus, respectively, with a 4 mm magic-angle-spinning (MAS) probe operating at a spinning rate of 12 kHz. ^{31}P MAS NMR spectra with high power proton decoupling were recorded using a π/2 pulse length of 3.3 μs and a recycle delay of 15 s. The chemical shifts of ^{31}P nucleus were externally referenced to adamantane, and $(NH_4)_2HPO_4$ (1 ppm), respectively. Prior to the NMR experiments, the synthesized 2D hybrid solid acids were performed as follows: before the sorption of probe molecules, the sample was placed into a glass tube and then connected to a vacuum line for dehydration at 180 ^oC. The temperature was gradually increased to 180 ^oC at a heating rate of 1 ^oC /min, and the system pressure was kept below 10^{-3} Pa over a period of 10 h and then cooled down to room temperature. Subsequently, sufficient volatile TMP molecule was transfer onto the sample frozen over a liquid N_2 bath. Then, the sample was evacuated at room temperature for 1 h to remove physisorbed TMP molecules after the adsorption was equilibrated. Finally, the sample tube was flame-sealed. The detailed procedures involved in introduction of TMPO probe molecule onto the sample are described into detail elsewhere.[S5-S7]. In brief, a certain amount of TMPO adsorbate dissolved in anhydrous CH_2Cl_2 was firstly added into a vessel containing the dehydrated sample in a N_2 glove box, then the CH_2Cl_2 solvent was removed from evacuation at room temperature. To

ensure a uniform adsorption of adsorbate probe molecules in the pores/channels of the synthesized 2D solid acids, the sealed vessel was further subjected to a thermal treatment at 160 ^oC for 2 h. Prior to NMR measurements, the sealed sample tube was opened and the sample was transferred into a NMR rotor with a Kel-F end cap under a dry nitrogen atmosphere in a glove box.

5. Hammett indicators characterization

In the typical procedure for determining the acidic strength, a 0.1 wt% Hammett indicator diluted in dry toluene was mixed with 0.1 g of the sample in a sealed sample vial. The resultant suspension was stirred at room temperature for 6 h, and the color changes of the indicator were recorded (Table S4).[S8,S9] The acidity of the solid samples were also measured by the potentiometric titration method. A 30 mg sample was dispersed in 50 mL of acetonitrile solvent and then stirred at room temperature for 6 h, then the suspension was titrated with 0.005 N n-butylamine in acetonitrile.[S10,S11]

6. Catalytic reactions

6.1. Esterification of oleic acid and methanol

Oleic acid (633 μL, 2 mmol), methanol (810 μL, 20 mmol), and TBA_6-P_2W_{17}-SO_3H (56.7 mg, 10 wt% based on the weight of oleic acid, 9.6×10^{-3} mmol) were added into a round-bottom flask and heated under reflux at 70 ^oC with vigorous stirring for 20 min. After that, the catalyst was separated by filtration, washed with methanol and dried under vacuum at 60 ^oC. The reaction conversion and yield were determined by gas chromatography.

6.2. Dehydration of the fructose to 5-HMF

The reaction mixture was purged with nitrogen to avoid the decomposition of 5-HMF. In a typical reaction, TBA_6-P_2W_{17}-SO_3H (150 mg) was added to the fructose (450 mg, 2.5 mmol) in 10 mL of 1,4-dioxane. Then, the reaction mixture was heated to 100 ^oC for 2 h. After that, the mixture was cooled gradually to room temperature, and the solid catalyst was separated simply by filtration.

For the recyclability test, the reaction mixture of fructose (450 mg, 2.5 mmol), catalyst (150 mg), and 10 mL of 1,4-dioxane at 100 ^{o}C for 2 h with N_2. The solid was separated by filtration, washed with methanol, dried at 70 ^{o}C under vacuum overnight and reused for each run.

6.3. Yield and selectivity

The oleic acid conversion (mol%), methyl oleate yield (mol%) and selectivity (mol%) were shown below:

$$Oleic\ acid\ conversion\ (mol\%) = 1 - \frac{Moles\ of\ oleic\ acid\ in\ product}{Starting\ amount\ of\ oleic\ acid} \times 100\%$$

$$Methyl\ oleate\ yield\ (mol\%) = \frac{Moles\ of\ methyl\ oleate\ in\ produced}{Starting\ amount\ of\ oleic\ acid} \times 100\%$$

$$Methyl\ oleate\ selectivity\ (\%) = \frac{Yield\ of\ methyl\ oleate}{Oleic\ acid\ conversion} \times 100\%$$

The fructose conversion (mol%), 5-HMF yield (mol%) and selectivity (mol%) were shown below:

$$Fructose\ conversion(mol\%) = 1 - \frac{Moles\ of\ fructose\ \ in\ product}{Starting\ amount\ of\ \ fructose} \times 100\%$$

$$5 - HMF\ yield\ (mol\%) = \frac{Moles\ of\ 5 - HMF\ in\ produced}{Starting\ amount\ of\ fructose} \times 100\%$$

$$5 - HMF\ selectivity\ (\%) = \frac{Yield\ of\ 5 - HMF}{Fructose\ conversion} \times 100\%$$

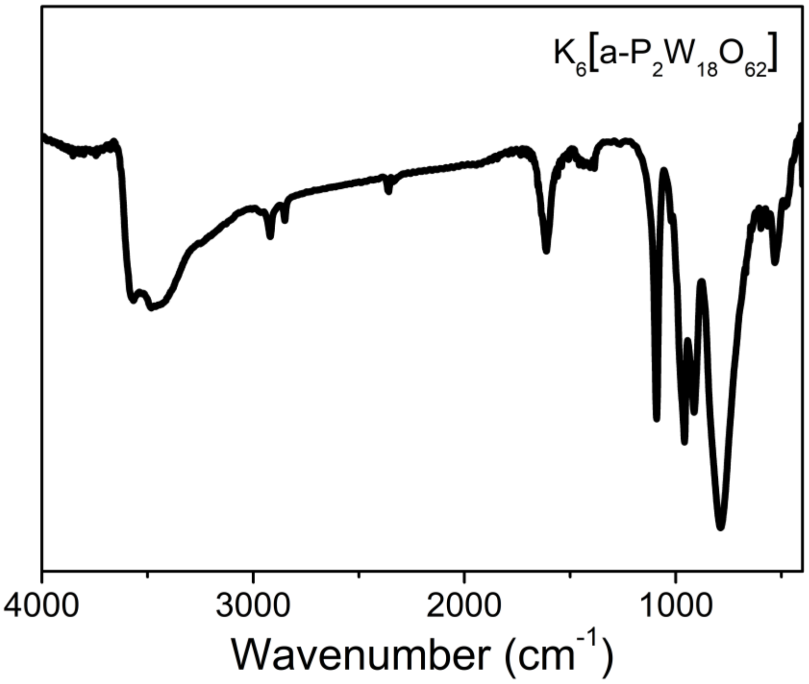

Figure S1. FT-IR spectrum of $K_6[\alpha\text{-}P_2W_{18}O_{62}]\cdot xH_2O$

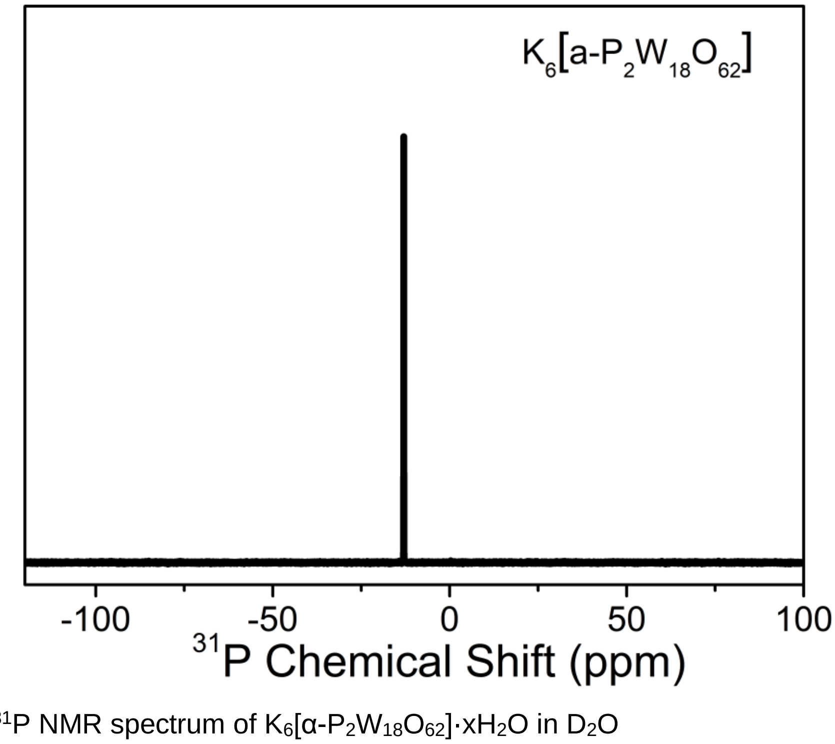

Figure S2. ^{31}P NMR spectrum of $K_6[\alpha\text{-}P_2W_{18}O_{62}]\cdot xH_2O$ in D_2O

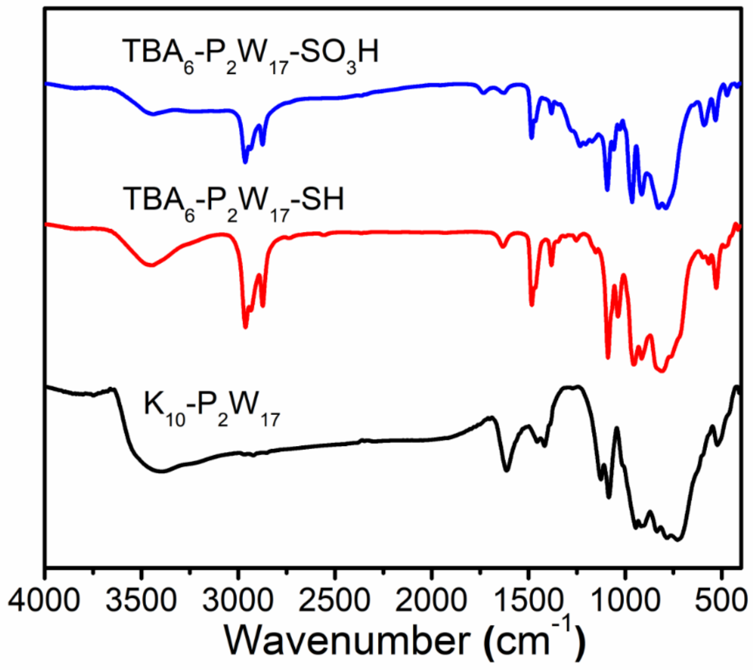

Figure S3. FT-IR spectra of K_{10}-P_2W_{17}, TBA_6-P_2W_{17}-SH and TBA_6-P_2W_{17}-SO_3H.

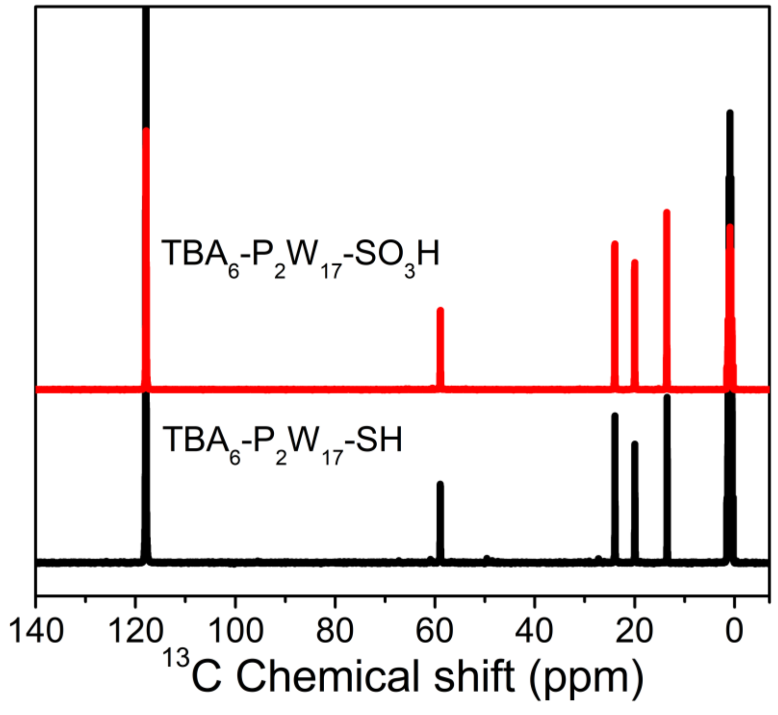

Figure S4. ^{13}C NMR spectra of TBA_6-P_2W_{17}-SH and TBA_6-P_2W_{17}-SO_3H in CD_3CN.

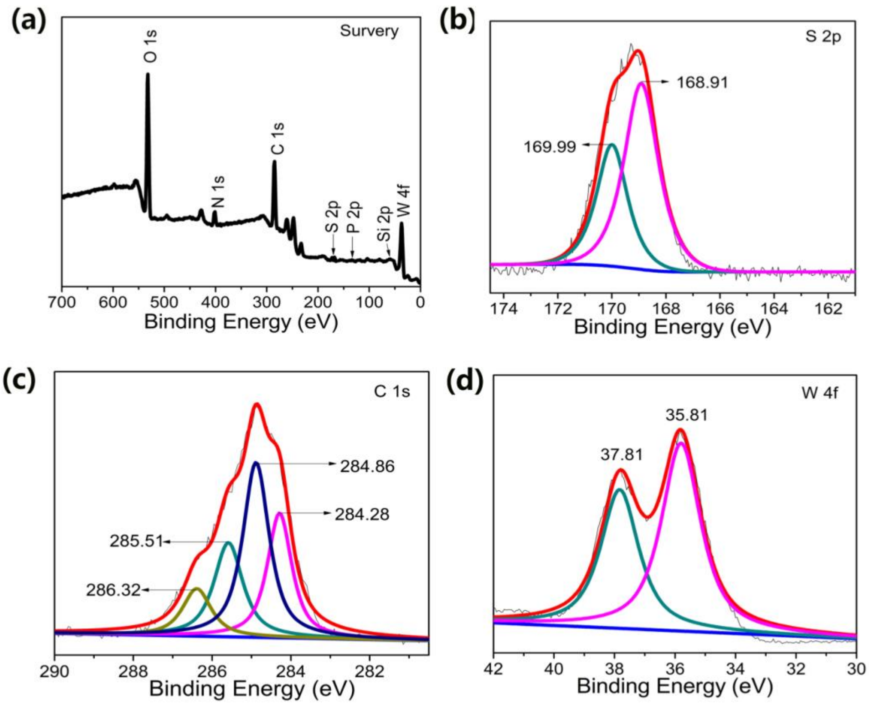

Figure S5. XPS curves of TBA_6-P_2W_{17}-SO_3H sample and XPS wide-scan spectra of S 2p, C 1s and W 4f.

Table S1. ESI-MS spectra of TBA_6-P_2W_{17}-SO_3H.

Entry	*m/z*	*z*	Molecular formula
**1**	2967.2	2-	$\{TBA_6[P_2W_{17}O_{62}(Si(CH_2)_3SO_3)_2]\}^{2-}$
**2**	2846.5	2-	$\{TBA_5[P_2W_{17}O_{62}(Si(CH_2)_3SO_3H)(Si(CH_2)_3SO_3)]\}^{2-}$
**3**	2726.0	2-	$\{TBA_4[P_2W_{17}O_{62}(Si(CH_2)_3SO_3H)_2]\}^{2-}$
**4**	1817.0	3-	$\{TBA_4[P_2W_{17}O_{62}(Si(CH_2)_3SO_3H)(Si(CH_2)_3SO_3)]\}^{3-}$
**5**	1736.6	3-	$\{TBA_3[P_2W_{17}O_{62}(Si(CH_2)_3SO_3H)_2]\}^{3-}$
**6**	1362.5	4-	$\{TBA_4[P_2W_{17}O_{62}(Si(CH_2)_3SO_3)_2]\}^{4-}$
**7**	1302.25	4-	$\{TBA_3[P_2W_{17}O_{62}(Si(CH_2)_3SO_3H)(Si(CH_2)_3SO_3)]\}^{4-}$
**8**	1242.0	4-	$\{TBA_2[P_2W_{17}O_{62}(Si(CH_2)_3SO_3H)_2]\}^{4-}$
**9**	993.4	5-	$\{TBA_2[P_2W_{17}O_{62}(Si(CH_2)_3SO_3H)(Si(CH_2)_3SO_3)]\}^{5-}$

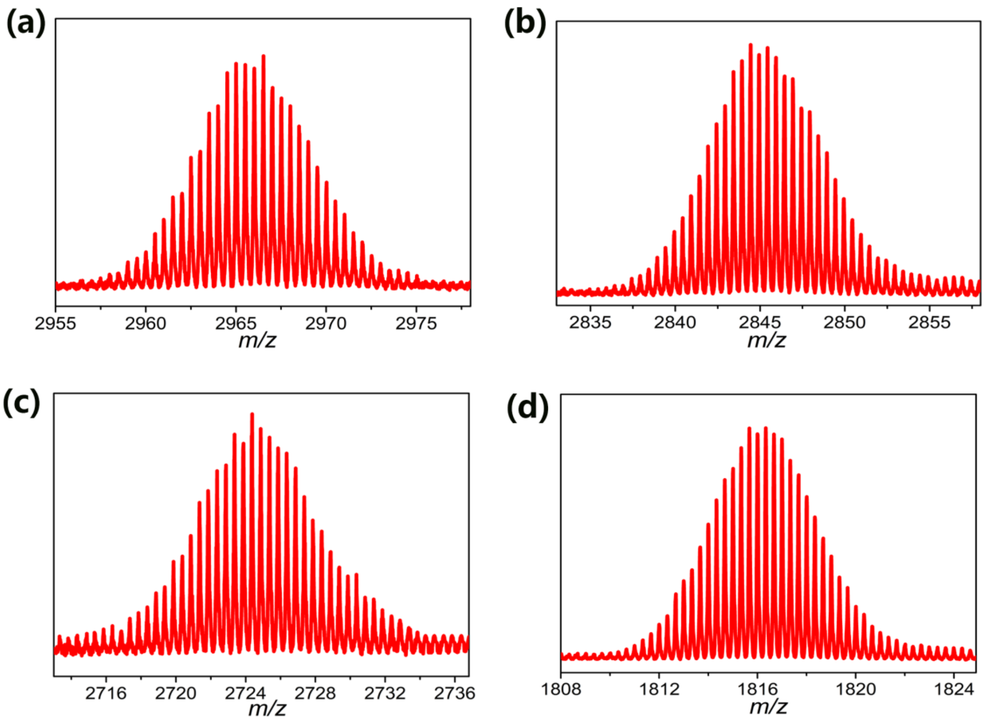

Figure S6. ESI-MS spectra of TBA_6-P_2W_{17}-SO_3H.

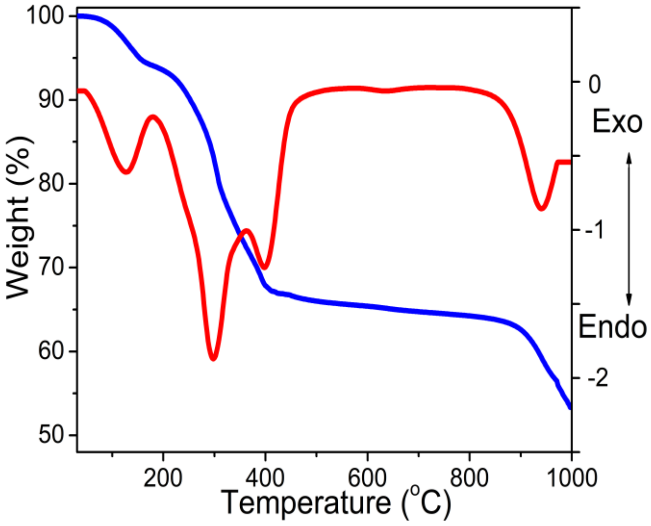

Figure S7. TG-DTA of TBA_6-P_2W_{17}-SO_3H.

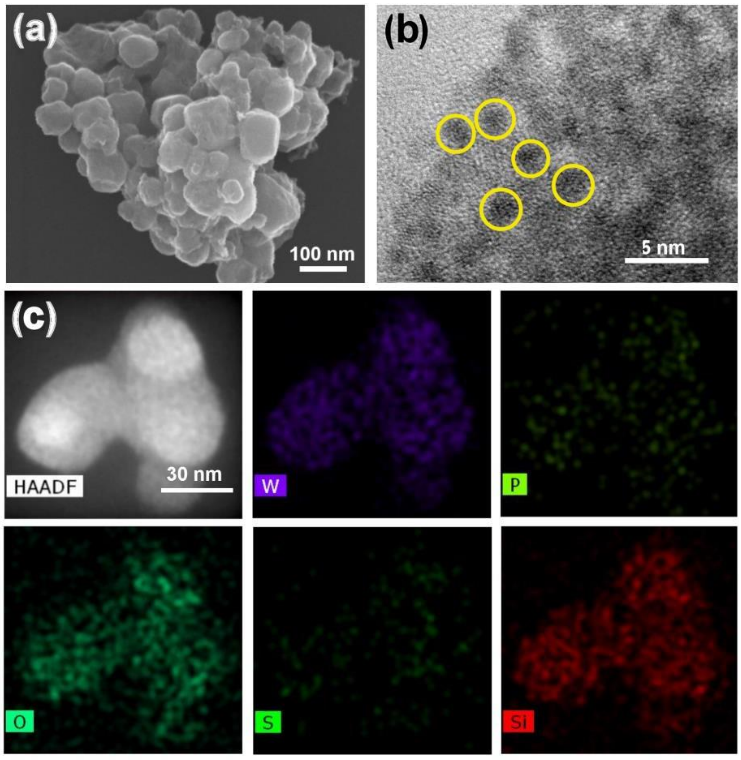

Figure S8. (a) SEM image, (b) HRTEM image and (c) HAADF images of TBA_6-P_2W_{17}-SO_3H.

Table S2. Hammett indicators and acidity of TBA_6-P_2W_{17} and TBA_6-P_2W_{17}-SO_3H measured by Hammett indicator tests.

**Indicators**	**Color[a]**		**pKa**	**Hammett Indicator Tests[b]**	
	Acid Form	Base Form		TBA_6-P_2W_{17}	TBA_6-P_2W_{17}-SO_3H
2-Nitroaniline	Red	Yellow	-0.2	-	+
2,4-Dinitroaniline	Red	Yellow	-4.4	-	+
2-Benzoylnaphthalene	Yellow	Colorless	-5.9	-	+
2-Bromo-4,6-dinitroaniline	Red	Yellow	-6.6	-	+
Anthraquinone	Yellow	Colorless	-8.1	-	+
4-Nitrotoluene	Yellow	Colorless	-11.4	-	+

[a]See ref S8, 9.

[b]Results of Hammett indicator tests are denoted as color change observed (+) and color change not observed (-).

Table S3. The acidity data on catalysts

Entry	Catalyst	Potentiometric titration, E_i (mV)	Total acid density (mmol H^+ g^{-1})
**1**	TBA_6-P_2W_{17}	12.4	0.07
**2**	TBA_6-P_2W_{17}-SH	7.9	0.10
**3**	TBA_6-P_2W_{17}-SO_3H	385.0	0.54

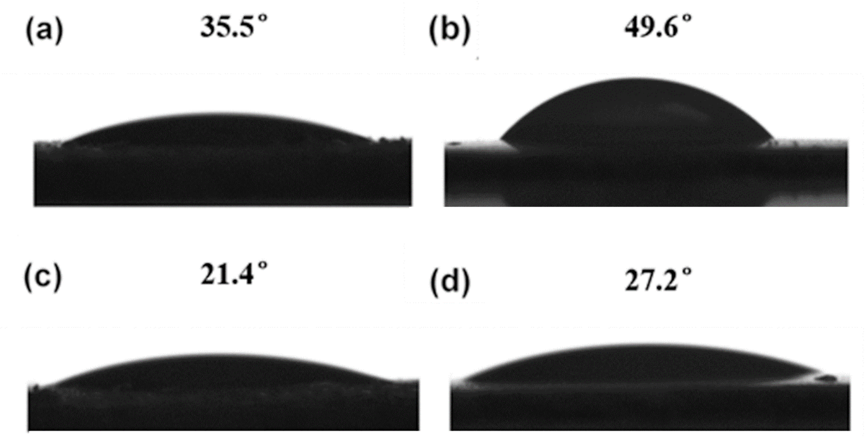

Figure S9. (a) Contact angles of an ethyl acetate droplet on the surface of TBA_6-P_2W_{17}-SO_3H; (b) Contact angles of an H_2O droplet on the surface of (a); (c) Contact angles of an H_2O droplet on the surface of TBA_6-P_2W_{17}-SO_3H; (d) Contact angles of an ethyl acetate droplet on the surface of (c).

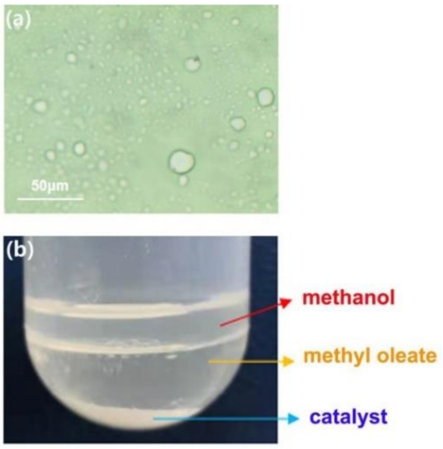

Figure S10. (a) Optical microgragh of a mixture of TBA_6-P_2W_{17}-SO_3H and oleic acid with methanol at 70 ^{o}C in 10 min; (b) Macroscopic views of MeOH/oleic acid stabilized by TBA_6-P_2W_{17}-SO_3H obtained after phase separation.

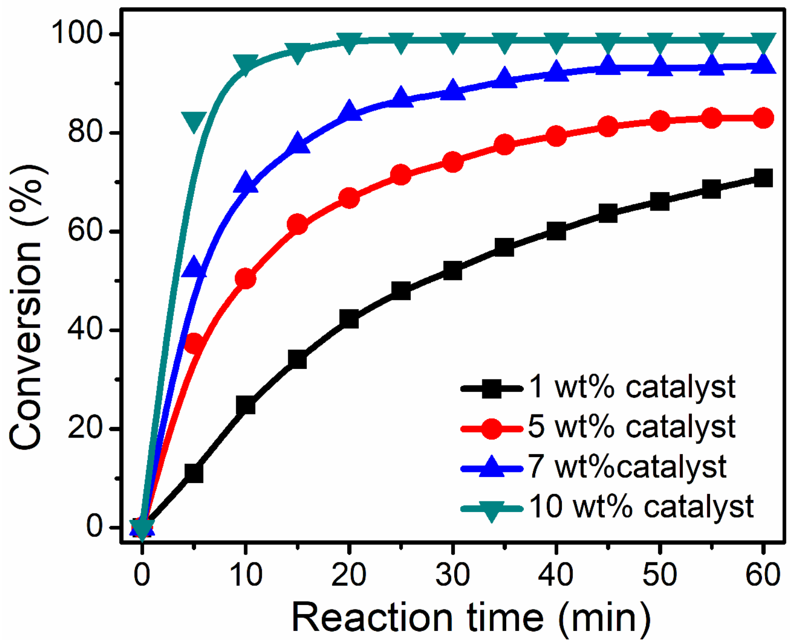

Figure S11. Influence of the catalyst dosage on the catalytic activity of oleic acid with methanol using the TBA_6-P_2W_{17}-SO_3H as catalyst at 70 ^oC. Reaction conditions: oleic acid 2 mmol, methanol 20 mmol, catalyst weight 1, 5, 7 and 10 wt% acid, t = 60 min, T= 70 ^oC.

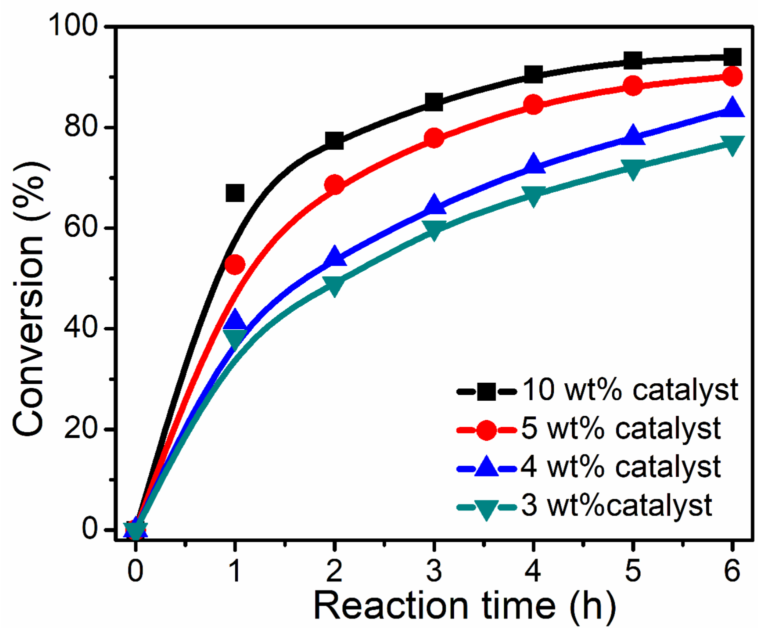

Figure S12. Influence of the catalyst dosage on the catalytic activity of of oleic acid with methanol using the TBA_6-P_2W_{17}-SO_3H as catalyst at 30 ^oC. Reaction conditions: oleic acid 2 mmol, methanol 20 mmol, catalyst weight 3, 4, 5 and 10 wt% acid, t = 6 h, T= 30 ^oC.

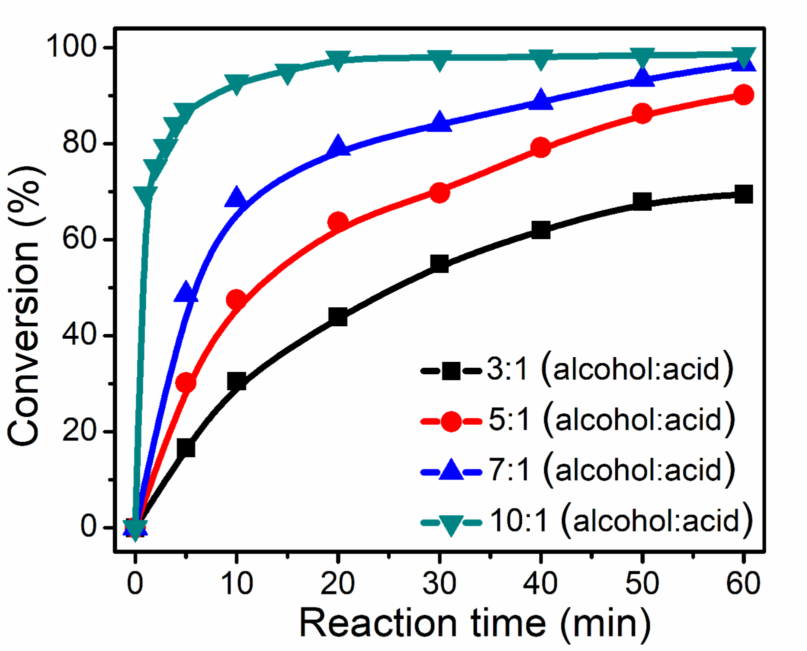

Figure S13. Influence of the ratio of oleic acid and methanol on the catalytic activity of oleic acid with methanol using the TBA_6-P_2W_{17}-SO_3H as catalyst at 70 ^oC. Reaction conditions: oleic acid 2 mmol, methanol 6, 10, 14 and 20 mmol, catalyst weight 10 wt% acid, t = 60 min, T = 70 ^oC

Table S4 Yield and selectivity of methyl oleate catalyzed by various acid catalysts.

Entry	Catalyst	Yield [%]	Sel. [%]
**1**	H_2SO_4[a]	88.24	99.0
**2**	$H_3PW_{12}O_{40}$[b]	67.71	99.0
**3**	$H_3PMo_{12}O_{40}$[b]	65.68	99.0
**4**	K_{10}-P_2W_{17}[b]	0.62	99.0
**5**	TBA_6-P_2W_{17}[b]	1.03	99.0
**6**	TBA_6-P_2W_{17}-SH[b]	0.70	99.0
**7**	TBA_6-P_2W_{17}-SO_3H[b]	98.67	99.0

[a]Reaction conditions: oleic acid 2 mmol, methanol 20 mmol, catalyst 6.0 mg (mol% same as TBA_6-P_2W_{17}-SO_3H), 70 ^oC.

[b]Reaction conditions: oleic acid 2 mmol, methanol 20 mmol, catalyst 56.7 mg, 70 ^oC.

Table S5. Results of various esterification reactions over the TBA_6-P_2W_{17}-SO_3H

Entry	Carboxylic acid	Alcohols	Yield [%]	T (min)
**1**	oleic acid	methanol	98.67	20
**2**	oleic acid	ethanol	99.23	30
**3**	oleic acid	propanol	96.75	65
**4**	oleic acid	butanol	97.12	75
**5**	oleic acid	pentanol	97.57	90
**6**	oleic acid	hexanol	99.47	100
**7**	oleic acid	heptanol	99.66	120
**8**	oleic acid	octanol	98.67	140
**9**	propionic acid	methanol	97.64	20
**10**	butyric acid	methanol	97.41	25
**11**	valeric acid	methanol	97.16	25
**12**	caproic acid	methanol	97.46	25
**13**	heptanoic acid	methanol	97.67	30
**14**	caprylic acid	methanol	97.33	30
**15**	lauric acid	benzyl alcohol	96.59	120
**16**	caproic acid	benzyl alcohol	97.62	120
**17**	5-hexinic acid	methanol	98.83	30
**18**	methacrylic acid	methanol	97.42	30

Reaction conditions: acid 2 mmol, alcohol 20 mmol, catalyst 10 wt% based on the weight of oleic acid, 70 ^{o}C.

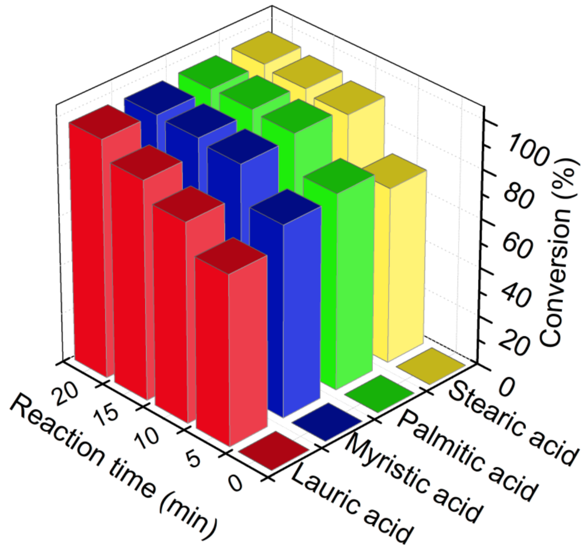

Figure S14. Esterification of long-chain acids and methanol. Reaction conditions: acid 2 mmol, methanol 20 mmol, catalyst 10 wt% based on the weight of acid, t = 20 min, T = 70 ^{o}C.

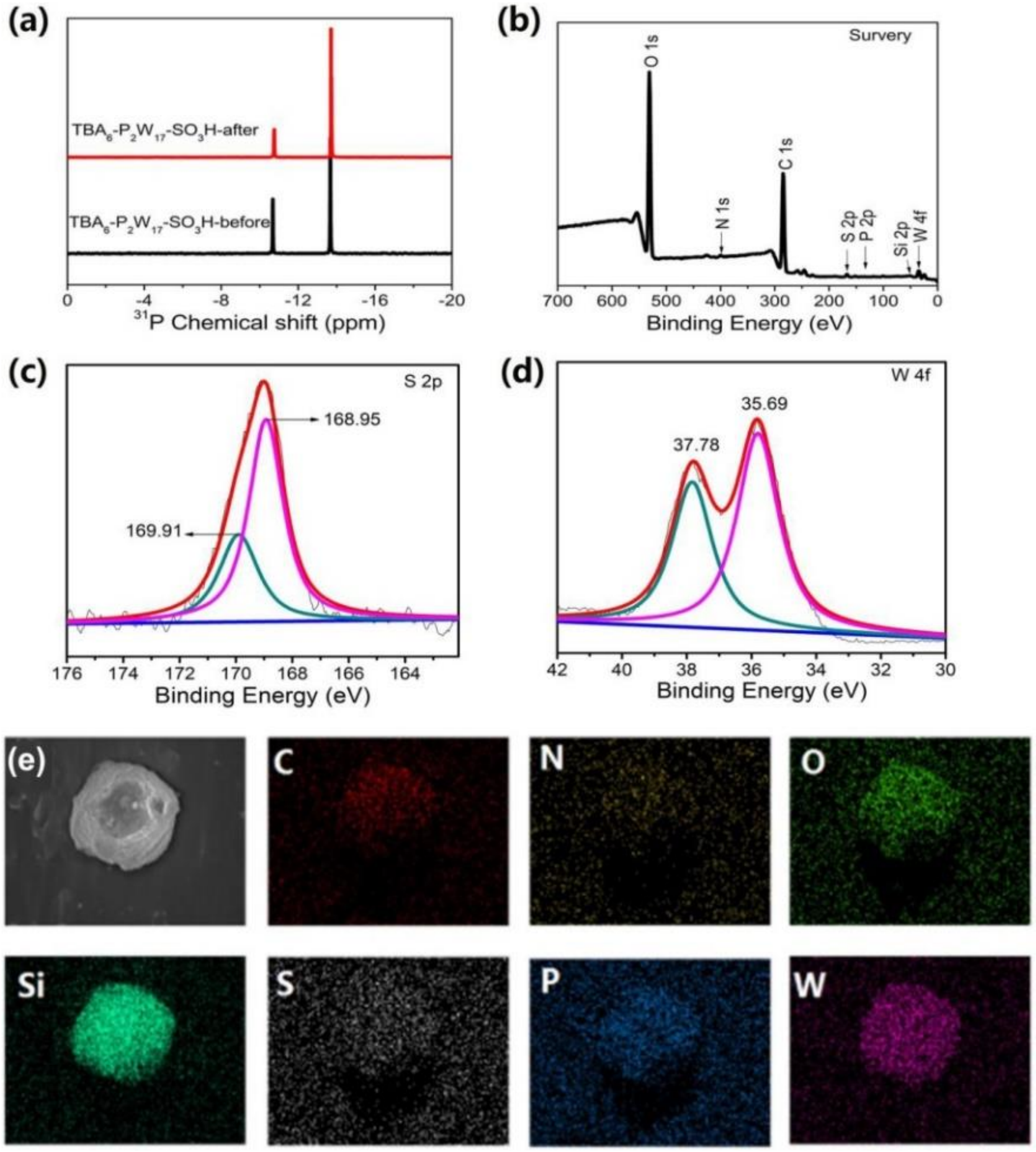

Figure S15. (a) ^{31}P NMR spectra, (b-d) XPS curves and XPS wide-scan spectra of S 2p and W 4f, (e) Elemental (C, N, O, P, Si, S and W) mapping of reused TBA_6-P_2W_{17}-SO_3H.

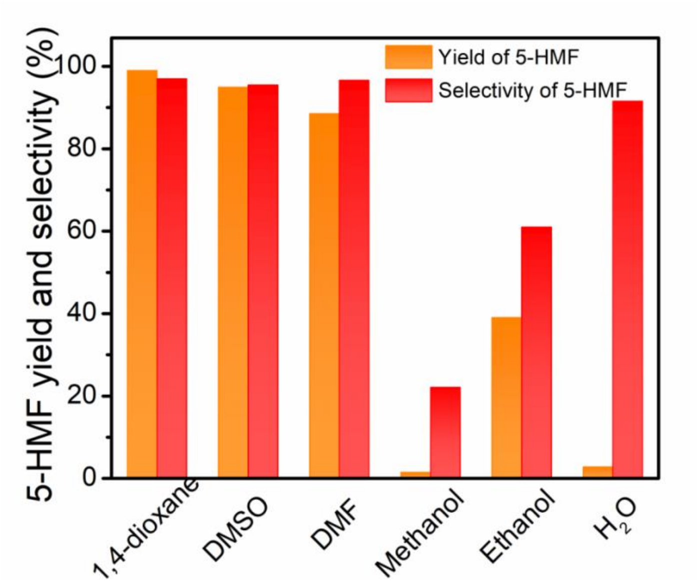

Figure S16. Influence of the solvents on the catalytic activity of fructose dehydration using the TBA_6-P_2W_{17}-SO_3H as catalyst. Reaction conditions: fructose 2.5 mmol; solvent 10 mL; the weight of TBA_6-P_2W_{17}-SO_3H 150 mg; 2 h; 100 ^oC; in N_2 atmosphere.

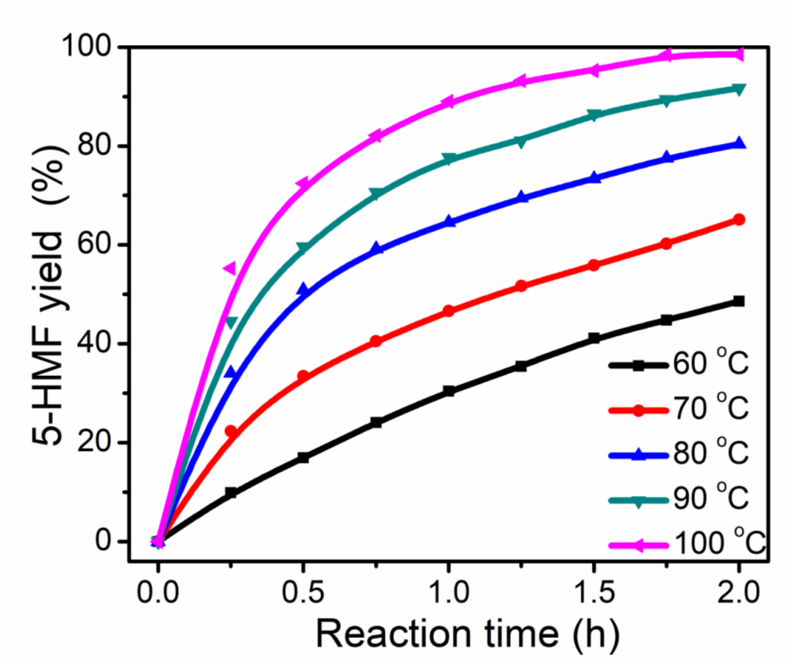

Figure S17. Influence of the temperature on the catalytic activity of fructose dehydration using the TBA_6-P_2W_{17}-SO_3H as catalyst. Reaction conditions: fructose 2.5 mmol; 1,4-dioxane 10 mL; the weight of TBA_6-P_2W_{17}-SO_3H 150 mg; 2 h; 60, 70, 80, 90 and 100 ^oC; in N_2 atmosphere.

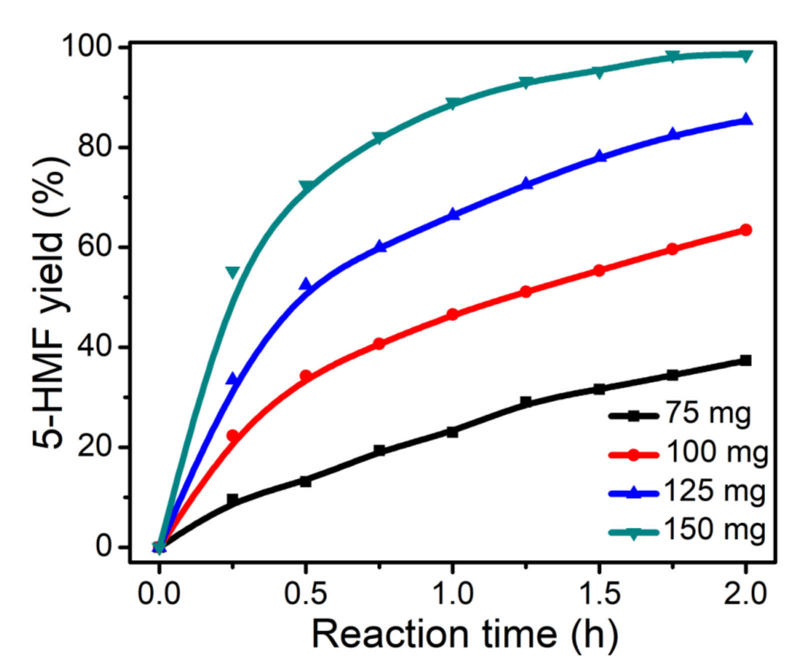

Figure S18. Influence of the catalyst dosage on the catalytic activity of fructose dehydration using the TBA_6-P_2W_{17}-SO_3H as catalyst. Reaction conditions: fructose 2.5 mmol; 1,4-dioxane 10 mL; the weight of TBA_6-P_2W_{17}-SO_3H 75, 100, 125 and 150 mg; 2 h; 100 ^oC; in N_2 atmosphere.

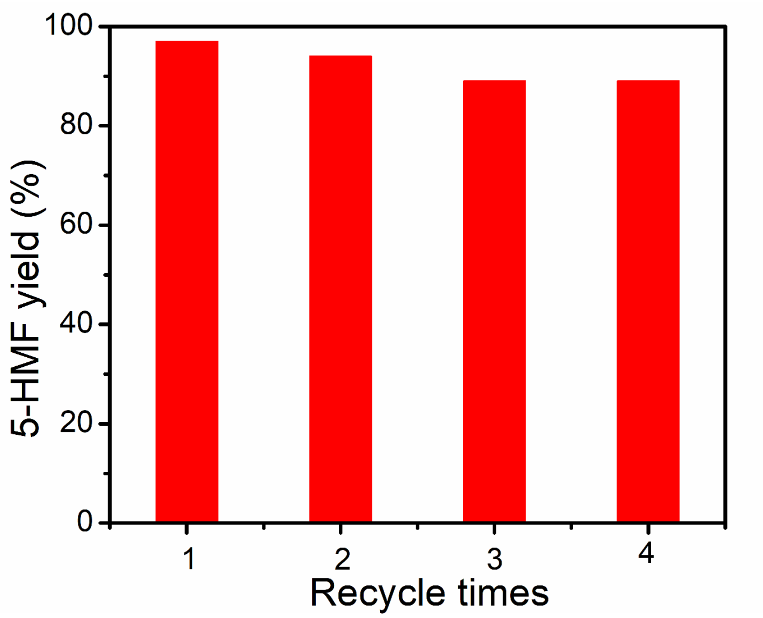

Figure S19. Recycling of TBA_6-P_2W_{17}-SO_3H.

References

[1] R. Contant, W. G. Klemperer, O. Yaghi, *Inorg. Synth.*, **2007**, *27*, 104-111.

[2] W. J. Randall, D. K. Lyon, P. J. Domaille, R. G. Finke, A. M. Khenkin, C. Hill, *Inorg.Synth.,* **1998**, *32*, 242-268.

[3] C. Boglio, G. Lenoble, C. Duhayon, B. Hasenknopf, R. Thouvenot, C. Zhang, R. C. Howell, B. P. Burton-Pye, L. C. Francesconi, E. Lacôte , S. Thorimbert, M. Malacria, C. Afonso, J.-C. Tabet, *Inorg Chem*., **2006**, *45*, 1389-1398.

[4] K. C. Nozaki, K. Yuhki, H. Kunihiko, Y. Asuka, H. Takeshi, N. Kenji, *Eur. J. Inorg. Chem.,* **2006**, *23*, 4834-4842.

[5] A. M. Zheng, H. L. Zhang, X. Lu, S.-B. Liu, F. Deng, *J. Phys. Chem. B.,* **2008**, *112*, 4496-4505.

[6] A. M. Zheng, S. J. Huang, S.-B. Liu, F. Deng, *Phys. Chem. Chem. Phys.,* **2011**, *13*, 14889-14901.

[7] N. Feng, A. M. Zheng, S.-J. Huang, H. L. Zhang, N. Y. Yu, C.-Y. Yang, S.-B. Liu, F. Deng, *J. Phys. Chem. C.,* **2010**, *114*, 15464-15472.

[8] J. C. Jiang, F. Gándara, Y. B. Zhang, K. Na, O. M. Yaghi, W. G. Klemperer. *J. Am. Chem.Soc.* **2014**, *136*, 12844-12847.

[9] C. C. Huang, C. J. Yang, P. J. Gao, N. Ci Wang, C. L. Chenb, J. S. Chang, *Green Chem.,* **2015**, *17*, 3609-3620.

[10] H. M. Altass, A. E. R. S. Khder, *J Mol Catal-Chem.,* **2016**, *411*,138-145.

[11] M. Kuzminska, T. V. Kovalchuk, R. Backo, E. M. Gaigneaux, *J. Catal.*, **2014**, *320*, 1-8.
